# Intestinal Autophagy and Its Pharmacological Control in Inflammatory Bowel Disease

**DOI:** 10.3389/fimmu.2016.00695

**Published:** 2017-01-09

**Authors:** Ping Ke, Bo-Zong Shao, Zhe-Qi Xu, Xiong-Wen Chen, Chong Liu

**Affiliations:** ^1^Department of Pharmacology, Second Military Medical University, Shanghai, China

**Keywords:** autophagy, Paneth cell, macrophage, goblet cell, inflammatory bowel disease, immune reaction

## Abstract

Intestinal mucosal barrier, mainly composed of the intestinal mucus layer and the epithelium, plays a critical role in nutrient absorption as well as protection from pathogenic microorganisms. It is widely acknowledged that the damage of intestinal mucosal barrier or the disturbance of microorganism balance in the intestinal tract contributes greatly to the pathogenesis and progression of inflammatory bowel disease (IBD), which mainly includes Crohn’s disease and ulcerative colitis. Autophagy is an evolutionarily conserved catabolic process that involves degradation of protein aggregates and damaged organelles for recycling. The roles of autophagy in the pathogenesis and progression of IBD have been increasingly studied. This present review mainly describes the roles of autophagy of Paneth cells, macrophages, and goblet cells in IBD, and finally, several potential therapeutic strategies for IBD taking advantage of autophagy.

## Introduction

The intestinal tract mainly functions as the most important organ digesting and absorbing food and nutrients taken orally. Besides those obvious functions, the gut is also regarded as one of the largest immune organs in an organism since the gut lumen always harbors a great number of microorganisms. As a result, the host has to maintain the peaceful coexistence with this diverse microbial community and trigger the inflammatory and immune reaction for the detection and elimination of the pathogenic microorganisms (1, 2). During this process, an efficient intestinal mucosal barrier is critical for the maintenance of microbial homeostasis and fighting against pathogenic microorganisms, providing the first line of defense. In general, the intestinal mucosal barrier is composed of two layers, including the intestinal mucus layer and the epithelium, in combination with the equipment of diverse specific and unspecific protective mechanisms which collectively build up an effective intestinal mucosal barrier (3). However, the disturbance of microbial homeostasis and damage of intestinal mucosal barrier largely trigger the inflammatory responses, immune reaction, accumulation of reactive oxygen species (ROS), and mitochondrial dysfunction in the intestinal wall, finally leading to the pathogenesis and progression of inflammatory bowel disease (IBD). As a result, inhibiting the over-activation of those self-defensive processes may serve as a potential and effective strategy for the treatment of IBD. Autophagy is a popular self-protective mechanism, the function of which mainly relies on lysosome. There is much evidence to suggest that autophagy could effectively attenuate the over-triggering of several self-defensive pathways such as inflammatory reaction and immune responses (4–6). Based on the statement, autophagy has been increasingly studied by researchers for the development of novel and effective therapeutic strategies against inflammation- or immune-related disorders, including IBD. Here, in this review, we discuss the mechanisms underlying the pathogenesis of IBD, the signaling pathways of autophagy, as well as the roles of autophagy in three important cells, namely, Paneth cells, macrophages, and goblet cells, in IBD, and, last but not least, several pharmacological interventions of autophagy in the treatment of IBD.

## Pathogenesis of IBD

We have mentioned above that the inflammatory and immune responses are triggered for the defense in the intestine. However, the over-triggered self-protective inflammatory and immune responses can lead to the damage of intestine itself as well as the intestinal mucosal barrier, contributing to the pathogenesis and progression of chronic inflammatory disorders, such as IBD.

Although the exact mechanisms underlying IBD have not yet been clarified, evidence has shown that the environment, genetics, and interactions between host defense and commensal microbiota are the main causes leading to the development of IBD (7). Smoking is an important environmental risk factor related to Crohn’s disease (CD) (8). Patients suffering from CD are responding badly to treatments and present more severe symptoms (9, 10). Another environmental factor attributing to IBD is air pollution (11). The particulate matter, ozone, or nitrous oxides in the air intrude into intestinal tract through food and water, which may cause an increased intestinal epithelial permeability, or induce pro-inflammatory response, thus leading to the occurrence of IBD (12, 13). In addition, it was noticed that antibiotics use was a potential factor of IBD onset as antibiotics could alter the amount and composition of the intestinal microflora, which ended up triggering abnormal inflammatory responses (14). The genetics of IBD were recognized by genome-wide association studies (GWAS) showing that people harboring variants in innate and adaptive immunity-related genes were more susceptible to both ulcerative colitis (UC) and CD, such as nucleotide-binding oligomerization domain-containing protein 2 (*Nod2*), immunity-related GTPase family protein M (*IRGM*), autophagy-related protein 16 like protein 1 (*Atg16l1*), interleukin 12B (*IL-12B*), *Drosophila* mothers against decapentaplegic protein 3 (*SMAD3*), and others (15–17). These susceptibility genes lead to dysregulation of the intestinal mucosal inflammatory and immune system that result in excessive immunologic responses to normal flora or impaired ability to clearance invasive bacteria (18, 19). Evidence has shown that virus-plus-susceptibility gene interactions induced colitis similar to CD (19). The interactions between the host and its diverse microbial community are complex and crucial in maintaining intestinal homeostasis. The intestinal defense system consists of the mucus layer, epithelium, and cells related to the innate immune system. In normal conditions, the mucus layer which contains large numbers of antimicrobial substance and mucins works together with the epithelium to separate microbes from the host, and if any pathogen intrudes through the epithelium, the dentritic cells and macrophages of the immune system in intestine will recognize it and generate an appropriate inflammatory or immune response by producing cytokines and antimicrobial agents (20). However, defects in mucin secretion (21) or epithelial integrity (22) and malfunction of the immune response to restrict pathogenic microbes alone or together contribute to the pathogenesis of IBD. The last factor for the pathogenesis and progression of IBD that deserves to be mentioned here is the adaptive immune response. It is generally acknowledged that increased pro-inflammatory cytokines produced by the T-helper cells or decreased anti-inflammatory cytokines induced by ineffective regulatory T-cells contribute to the pathogenesis of IBD (23, 24). Nevertheless, since the pathogenesis of IBD is complex and the exact underlying mechanisms still remain unclear, no effective therapeutic strategies have been developed for the treatment of IBD.

## Autophagy and Its Functions

Autophagy is an evolutionarily conserved catabolic process that involves degradation of protein aggregates and damaged organelles for recycling (25). Its function of degrading and recycling mainly relies on lysosome. In general, autophagy is defined into three types: macroautophagy, microautophagy, and chaperone-mediated autophagy (26, 27). Microautophagy belongs to a non-selective degradative mechanism through invagination of the lysosomal/vacuolar membranes for the engulfment of cytoplasmic components (28). Chaperone-mediated autophagy is the only autophagy style that allows the degradation of organelles and proteins relying on the presentation of chaperones. It has been reported previously that chaperone-mediated autophagy largely demands the presence of a targeting motifin which is the substrate protein, a set of cytosolic and lysosomal chaperones, and a receptor protein at the lysosomal membrane for the transportation into lysosome assisted by chaperone located in the lysosomal lumen (29). Macroautophagy is a catabolic process, during which intracellular components or invasive bacteria are surrounded by double-membrane-bound structures, widely acknowledged as autophagosome. After the confusion of autophagosome and lysosome, the degradative autolysosome with single-layer membrane is formed for the digestion of ingredients by lysozymes (30, 31). Since macroautophagy is, so far, the best-studied autophagy process, this review will mainly discuss the functions and roles of macroautophagy in IBD (hereafter referred to as “autophagy”).

In general, the induction of autophagy process is mainly involved in two steps. In the first step, the cup-shaped double-layer phagophores form in the cytoplasm, followed by the subsequent formation of spherical double-membraned autophagosomes that enclose misfolded proteins or damaged organelles for degradation. Autophagosomes are regarded to be shaped from the nucleation and membrane expansion of phagophores. In the second step, autophagosomes dispose of “coat proteins” named autophagy-related light chain 3 (LC3) on their surface followed by the integration with lysosomes for the formation of the single lipid layer membrane-surrounded autolysosomes, which are the functional units for degradation and digestion (32). The whole process is participated by more than 30 kinds of autophagy-related genes (Atgs) and autophagy-related proteins (32, 33).

So far, several signaling pathways have been uncovered for the involvement of autophagy process. As recently reviewed by us (32), there are two major signaling pathways participating in the induction and regulation of autophagy process, including the inhibitory Class I PI3K–mTOR signaling pathway and the inductive Class III PI3K–Beclin-1 signaling pathway. The inhibitory pathway is often activated by nutrient sufficiency or several growth factors stimulation. In this pathway, the Class I PI3K–mTOR signaling is largely activated by the phosphorylation of Akt pathway and formation of mTOR complex 1, a major anti-autophagy complex, which in turn induces the inhibition of Atg1 (ULK1) to prevent the initiation of autophagosome formation. The inductive signaling pathway often occurs in the opposite situations like nutrient insufficiency or stimulation of the inflammatory or ROS stress. In this process, the Class III PI3K–Beclin-1 complex is initially formed, which in turn promotes the assembly of the Atg12–Atg5–Atg16L complex and the Atg8/LC3. The triggering of this signaling pathway largely induces the formation of autophagosomes and autophagy process.

In the fundamental studies on autophagy, the application of autophagy inhibitors is commonly conducted as an effective strategy in the exploration of autophagy mechanisms and discussion on roles of autophagy in diseases. Traditionally, chemical inhibitors of autophagy were most widely used. According to the latest guidelines for the use and interpretation of assays for monitoring autophagy published in 2016, the chemical inhibitors of autophagy can be mainly divided into two categories based on the stages of autophagy blockade (34). The first one, including 3-methyladenine (3-MA), LY294002, and wortmannin, belongs to sequestration inhibitors, relying on the inhibition of PI3Ks as well as class III PtdIns3Ks, thus leading to the blocking of the formation of autophagosomes. The second one, including vinblastine, leupeptin, and bafilomycin A_1_, is post-sequestration inhibitors, which leads to the accumulation of sequestered materials in either autophagosomes or autolysosomes or both. However, researchers are increasingly aware that most chemical inhibitors are not entirely specific and show the dose- and time-dependent effects. For example, it was reported that in a certain condition for the application of 3-MA, autophagy might be promoted because of the inhibition of the class I enzyme (35). Recently, the specific loss-of-function *Atg* mutants are considered as the more effective approach for the blockade of autophagy process, but they may lead to the autophagy-independent effects and also be dispensable of autophagy (34). Consequently, more efforts must be made in order to seek the more effective and specific autophagy inhibitors.

So far, autophagy has been reported to be closely related with apoptosis, inflammatory response, immune reaction, ROS stress, and mitochondrial dysfunction, functioning as effective regulator of the occurrence and extent of those processes (31, 36–38). As a result, it is certain that autophagy is a vital factor in the pathogenesis and regulation of various kinds of inflammation- and immune-related diseases, serving as a potential and effective target for the intervention of those diseases.

## The Roles of Autophagy in IBD

The roles of autophagy in the pathogenesis and progression of IBD has been increasingly studied by researchers recently. For example, GWAS have reported several Atgs like *Atg16l1* and *IRGM* contributes to the susceptibility of IBD, suggesting that autophagy possibly mediated the pathophysiology of IBD (17, 39, 40). In fact, autophagy affects the pathogenesis of IBD in various pathways, one of which is to regulate the clearance of invading pathogens. When the host cells were infected by bacteria, cytoplasmic vesicles engulfed these pathogens to form autophagosome, thus confining them obtaining nutrients and promoting acidification of surroundings. The enhancement of autophagy promotes the integration of autophagosome and lysosome, leading to the degradation of pathogenic organisms (41–43). In addition, autophagy protects cells against the damage of bacterial toxins, thus promoting cell survival such as macrophages, neutrophils, and intestinal epithelial cells (44). It has also been reported that autophagy augments adaptive immune response. After degraded by autolysosome, antigens are presented to the major histocompatibility complex class II molecules (MHC II), and then recognized by T cells to prime the adaptive immune response. This process can be promoted by autophagy (45–47). Evidence has shown that impaired autophagy disturbs the function of intestinal epithelial cells and influences the innate and adaptive immune responses, ROS production, and endoplasmic reticulum (ER) stress, leading to abnormal inflammatory reaction, and ultimately promoting the occurrence and development of IBD (7, 48–50).

Moreover, it should be mentioned there exists the crosstalk between autophagy and the damage-associated molecular pattern molecules (DAMPs) release and degradation in IBD. DAMPs are defined as several kinds of endogenous molecules including the S100A calgranulins, chromatin-associated high-mobility group box 1 (HMGB1), heat shock proteins, interleukin (IL)-1 family members, histones, and adenosine triphosphate (ATP), DNA, RNA, uric acid, hyaluronan, and heparin sulfate which are released by dead, dying, injured, or stressed cells (51). Previous studies reported that high levels of DAMPs were detected in serum, fecal or mucosa of IBD patients as well as mice models (52–55). In addition, it was demonstrated that the regulation of IBD progression by DAMPs was majorly through several approaches: (1) affecting epithelial barrier function; (2) binding with pattern-recognition receptors (PRRs) to exert directly pro-inflammatory effect; and (3) assisting in antigen-presenting cells to regulate T cells function (56). Since DAMPs are highly relevant to IBD, it is extremely essential to monitor the release pattern of these molecules. It has been demonstrated that the levels of DAMPs can be decreased by autophagy through the promotion of DAMPs degradation (57–59). However, it was also noted that under certain conditions, such as starvation or the stimulation of cytotoxic drugs, the induced autophagy could substantially lead to the release of DAMPs, including HMGB1, ATP, and IL1B (60–62). Consequently, to ultimately take advantage of the inhibitory roles of autophagy on DAMPs, effective methods are demanded to get rid of the promoting effect of autophagy on the release of DAMPs.

The following parts of this paper will mainly focus on the influence of autophagy in three important kinds of cells, which are highly related to intestinal self-defense and inflammatory and immune reaction, namely Paneth cells, macrophages, and goblet cells, on IBD, and finally, several potential therapeutic strategies for the treatment of IBD taking advantage of autophagy.

### Paneth Cell, IBD, and Autophagy

#### Paneth Cell and IBD

As mentioned above, the epithelium together with mucus layer forms a physical barrier against the invading pathogens, including bacteria, fungi, virus, and harmful antigens in food, while tolerating beneficial microbes, thus maintaining the intestinal homeostasis. Specifically speaking, intestinal mucus layer, a layer of sticky gel covering the surface of mucosa, is composed of proteins, lipids, and carbohydrates as well as the large amount of water (63, 64). In the colon or large intestine, the mucus layer is divided into two distinct sectors, including the inner and outer layers. The inner layer is densely and firmly attached to the epithelium, while the outer layer is relatively more removable (3, 65). Among all of the components of mucus layer, various kinds of antimicrobial peptides (AMPs) are the most important functional peptides, with the defensins and the cathelicidins as the two best featuring families of AMPs, protecting the intestinal against microorganisms (66). The epithelium is majorly composed of several kinds of intestinal epithelial cells, including absorptive enterocytes, goblet cells, enteroendocrine cells, and Paneth cells (67). The absorptive enterocytes are the most abundant cell type in the epithelium, since they are rich in intestinal villi (68). Goblet cells and enteroendocrine cells mainly functions in secretion and restoration of the intestinal tissue (2, 68). Paneth cells, named after the Austrian physiologist Joseph Paneth, are the target cells in this paper for discussion, since they contains various kinds of secretary granules with antimicrobial active substances like lysozyme and α-definsins, highly involved in the defensive inflammatory and immune response in the intestine as well as the pathogenesis and progression of chronic intestinal diseases (3, 69). A recent study demonstrated that Paneth cells were a site of origin for intestinal inflammation, which are situated in the base of crypts of Lieberkühn, harboring a large number of ER and Golgi apparatus. Studies conducted on them revealed their vital role in directing the balance between homeostasis and inflammation through releasing AMPs and peptides including lysozyme, lipopolysaccharide (LPS)-binding protein, matrix metalloproteinase-7, phospholipase A2, phospholipase B, and IgA, as well as inflammatory cytokines like transforming growth factor β1 (TGF-β1), tumor necrosis factor α (TNF-α), and prostaglandin E2 (70–72). These AMPs could regulate the composition and quantity of microbes that colonize in the intestinal lumen and clear the intracellular pathogens.

#### The Roles of Paneth Cell Autophagy in IBD

In recent years, researchers discovered that autophagy and the autophagy gene had a specific role in the biology and function of Paneth cells. Cadwell et al. revealed that *Atg16l1*-, *Atg5*-, and *Atg7*-deficient Paneth cells exhibited few granules and decreased amounts of antimicrobial proteins and peptides inside. These cells also defected in granule exocytosis pathway through which cytoplasmic granules containing AMPs and other proteins are secreted to the intestinal lumen. In addition, *Atg16l1*-deficient Paneth cells presented increased expression of lipoprotein lipase, apolipoprotein A-IV, adiponectin, leptin, adiponectin, resistin-like α, complement factor D (adipsin), and haptoglobin. Many of these genes were directly implicated in inflammation, and two of these transcripts, namely leptin and adiponectin, increased in CD patients. Likewise, CD patients who carried *Atg16l1* risk allele showed morphology and granule abnormalities of Paneth cells similar to those observed in autophagy-gene-deficient mice and expressed increased levels of leptin protein (73–75). Different from decreased amounts of granules in *Atg7*-deficient Paneth cells reported by Cadwell et al., Wittkopf et al. found out that *Atg7* deficiency induced increased number and smaller size of granules in Paneth cells. Despite the lack of Paneth cell biology, *Atg7* deficiency did not lead to altered response toward dextran sodium sulfate (DSS)-induced colitis, suggesting that autophagy defects in intestinal epithelial cells alone was not potent enough to increase susceptibility to enteritis (76). This conclusion was further supported by Cadwell et al. who discovered that *Atg16l1* mutation in Paneth cells alone does not induce more severe injury in colon of DSS-induced colitis but a specific virus-plus-*Atg16l1* variant led to intestinal lesion in mice through secreting high levels of TNF-α and interferon-γ (IFN-γ) (19). Furthermore, global knockout of *Atg4b* caused alterations in Paneth cell, including less number and small size of granules and decreased lysozyme, and *Atg4b*-deficient mice were more susceptible to DSS-induced colitis (77). In addition to autophagy-related genes, some other molecules can regulate the level of autophagy in intestinal epithelial, like vitamin D receptor (VDR). The study showed that intestinal epithelial VDR deletion impaired antimicrobial function of Paneth cells by downregulation of *Atg16l1* and lysozyme, thus causing increased susceptibility to DSS-induced colitis (78). Additional IBD risk gene, *Nod2* or leucine-rich repeat kinase 2 (*LRRK2*) absence disturbs the lysozyme packaging and secretion of Paneth cells, thus leading to the failure in controlling pathogen invasion, which was shown by infection with *Listeria monocytogenes* (79).

**Figure 1:**
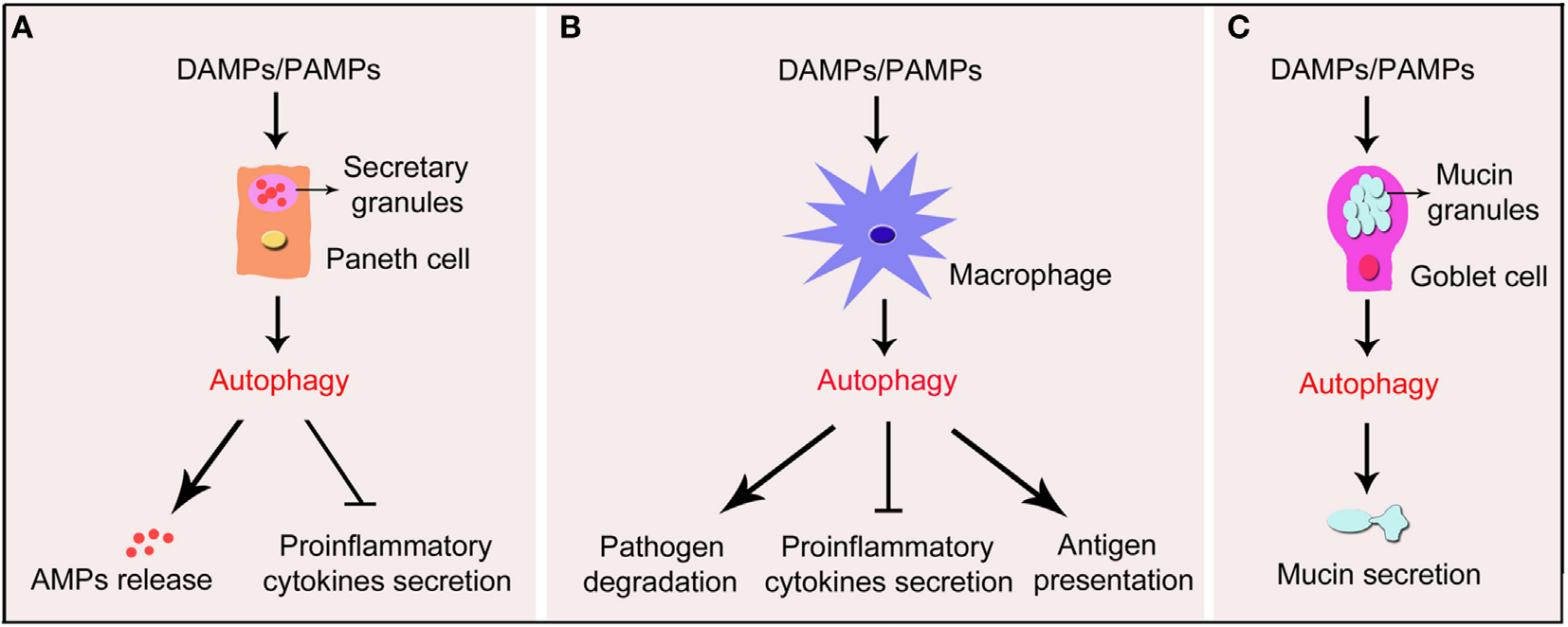
**Schematic illustration of impact of autophagy in Paneth cells, macrophages, and goblet cells in IBD**. Under the exposure of DAMPs or PAMPs, autophagy process is induced in Paneth cells, macrophages, and goblet cells in the gut wall. **(A)** Autophagy in Paneth cells triggers the formation of functional granules and substantial release of the AMPs. In addition, autophagy suppresses pro-inflammatory cytokines secreted by Paneth cells. **(B)** In macrophages, autophagy promotes the degradation of pathogens and the presentation of antigens. Autophagy also inhibits the secretion of pro-inflammatory cytokines by macrophages. **(C)** In goblet cells, autophagy promotes the formation of mucins granules and secretion of mucins. IBD, inflammatory bowel disease; DAMPs, damage-associated molecular pattern molecules; PAMPs, pathogen- associated molecular patterns; AMPs, antimicrobial peptides.

It is well known that dysfunction of ER causes unfolded and misfolded proteins accumulating in the ER lumen, which is called ER stress (80). ER stress disturbs the normal development and functions of cells, especially Paneth cells, because they are the IEC subtypes with the strongest function of secretion, which synthesize and excrete mounts of AMPs (81). As shown in recent discussion showed (82), there was a crosstalk between ER stress and inflammatory reaction. They noted that ER stress-induced inflammation in Paneth cells, epithelial cells, and goblet cells could possibly contribute to the pathogenesis and progression of IBD. However, they also argued that ER stress-induced inflammation could impede tumorigenesis through the induction of immunogenic cellular death-based antitumor immune responses despite its already demonstrated contribution of tumorigenesis, thus indicating the complexity of relationship between ER stress-induced inflammation and cancer. It was recently demonstrated that autophagy could be induced by ER stress in Paneth cells. The augmentation of autophagy in Paneth cells attenuates the ER stress-induced intestinal inflammation and eases nuclear factor-kappaB (NF-κB)-induced inflammatory reaction (72). This study uncovers the connection between autophagy and ER stress in the occurrence of IBD. In addition, it was reported that the ROS-mediated antibacterial autophagy (well-known as “xenophagy”) as well as the mitochondrial autophagy (well-known as “mitophagy”) in Paneth cells contributed greatly to the attenuation of IBD, thus probably serving as potential strategies for the treatment of IBD (83, 84). Collectively, these findings indicate that autophagy in epithelial cells, especially in Paneth cells, contributes to the alleviation of IBD (illustrated in Figure 1A).

### Macrophage, IBD, and Autophagy

#### Macrophage and IBD

The intestinal mucosa holds the largest population of macrophages in the body, which are vital and essential for the maintenance of mucosal homeostasis and epithelial renewal as well as protection against pathogenic microorganisms (85, 86). For the ontogeny of the gut-resident macrophages, it was traditionally believed that the majority of tissue macrophages were derived from the mononuclear phagocyte system in the bone marrow (87). So far, it is widely acknowledged that macrophages of the gut wall share the markers, including CD11c and MHC II, with other antigen-presenting cells like dendritic cells as well as “lineage-specifically” expressed CX3C chemokine receptor 1, F4/80, and CD64 (88, 89). Normally, macrophages are divided into M1 and M2 subtypes, the former displays pro-inflammatory characteristics and the later one has immunosuppressive and wound-healing properties (90). It was demonstrated that the gut-resident macrophages hold the features of both M1 and M2 subtypes. For instance, they have been reported to produce and secrete both the pro-inflammatory cytokines like TNF-α and the anti-inflammatory cytokines like IL-10, to adapt themselves to the environment of the gut wall (85, 91).

In the steady state, the gut-resident macrophages play an important role in the safeguarding of the intestine through the recognition and clearance of invading microorganisms and apoptotic cells as well as the regulation of tissue remodeling (92). The depletion of macrophages leads to the increasing susceptibility of DSS-induced colitis in mice (93). Intestinal macrophages can engulf and present the invading microorganisms to other immune cells like T cells, triggering the occurrence of immune reaction. Macrophages can also produce and secrete several pro-inflammatory cytokines and chemokines, including TNF-α, IL-6, IL-1β, and IL-12, leading to the further cascade amplification of inflammatory reaction (88, 94). It was demonstrated that during the occurrence of DSS-induced colitis in mice model, a large extent of infiltration of pro-inflammatory monocytes and macrophages was observed, leading to the further triggering of inflammatory and immune reaction, thus indicating that the regulation of macrophage-mediated inflammatory and immune responses might be vital in the treatment of IBD (94).

#### The Roles of Macrophage Autophagy in IBD

The roles of macrophage autophagy are generally illustrated in Figure 1B. It has been proven that pathogenesis and progression of IBD is highly related to the constant and overwhelming activation of innate immune reaction in gut wall, leading to the secretion of pro-inflammatory cytokines and chemokines, thus resulting in mucosal damage (95). Macrophages are regarded as important innate immune cells, since macrophages sense pathogen-associated molecular patterns of microorganisms and DAMPs through PRRs to initiate rapid and effective innate immune response to protect organism against pathogens along with DCs (96). What’s more, macrophages recognize antigens and present them to T cells to induce adaptive immune response (97). Given the key role of the interactions between host and microbe in the intestine, it is critical to regulate PRR signals and cytokine secretion properly. The NOD-like receptors (NLRs) in cytoplasm and Toll-like receptors (TLRs) on the surface are the two main types of PRRs in innate immune cells (95). It has been demonstrated that NLRs and TLRs in macrophages as well as other innate immune cells are closely associated with autophagy, and macrophage autophagy is highly related to the mediation of innate immune response in the gut wall (98, 99). For example, activating NOD2 using muramyldipeptide, a constituent of the bacterial peptidoglycan, recruited the autophagy-related protein ATG16L1 to the plasma membrane at the bacterial entry site to promote autophagy, but could not induce autophagy in cells with the *Atg16l1* risk variant, thus indicating a functional connection between *Nod2* and *Atg16l1* (100–102). In addition, it was reported that macrophages with CD-associated *Nod2* variants failed to induce autophagy, and the ability to kill pathogenic bacteria was impaired (103). Furthermore, there are much evidence of the connection between macrophage autophagy and the innate immune reaction in IBD. For instance, it was reported that impaired autophagy in macrophages transfected with siRNA targeted on *Atg16l1* or *IRGM* and peritoneal macrophages from *Nod2*-deficient mice increased the amounts of intracellular adherent-invasive *Escherichia coli* (AIEC) and the levels of IL-6 and TNF-α stimulated by AIEC (104). Moreover, downregulation of autophagy caused by *Atg4b* deficiency increased the susceptibility to DSS-induced colitis, which is not only through affecting the function of Paneth cells but by promoting the production of pro-inflammatory cytokines in colon tissues from mice induced by DSS and in peritoneal macrophages stimulated with LPS (77). Those studies indicate that autophagy deficiency leads to the enhancement of the innate immune response and the production of pro-inflammatory cytokines in the gut wall with the inflammatory stress, thus demonstrating the close association between macrophage autophagy and the innate immune response in IBD.

**Figure 2:**
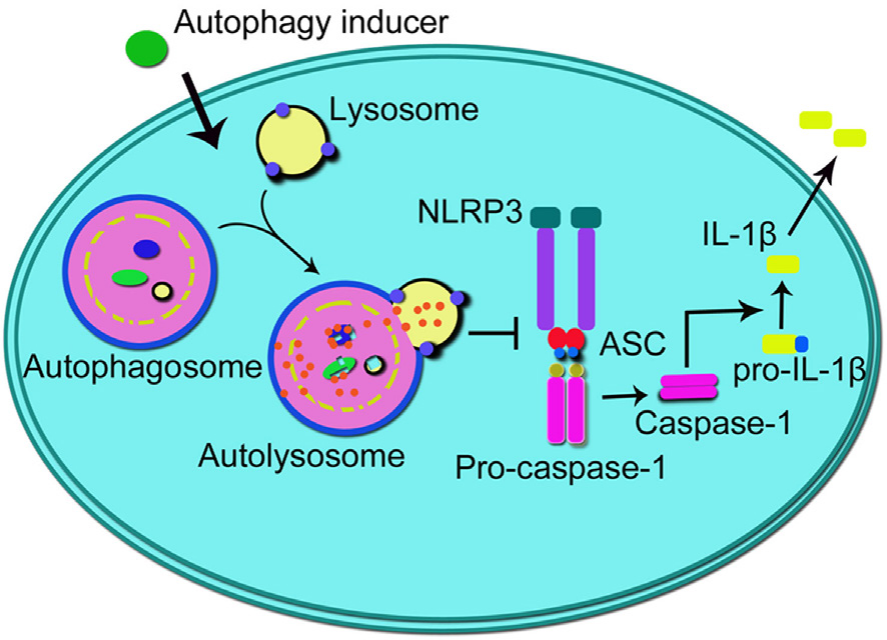
**Schematic illustration of the impact of autophagy on NLRP3 inflammasome**. Autophagy inducers promote autophagy process which inhibits the formation of NLRP3 inflammasome (integrated by NLRP3, ASC, and pro-caspase-1), thus suppressing the activation of caspase-1 and subsequent production of IL-1β. NLRP3, NLR family, pyrin domain-containing 3; ASC, adapter protein apoptosis-associated speck-like protein; IL-1β, interleukin-1β.

Recently, a member of the NLR family, namely NLR family, pyrin domain-containing 3 (NLRP3) inflammasome, has been implicated in the pathogenesis and progression of IBD (105–108). Among all kinds of inflammasomes, the NLRP3 inflammasome is the best characterized one, containing NLRP3 protein, adapter protein apoptosis-associated speck-like protein, and pro-caspase-1 (109, 110). Interactions among these three proteins tightly regulate inflammasome functions through inducing the production and secretion of IL-1β and IL-18, whose maturity is triggered by caspase-1 from their “pro” forms (110). It has been reported that the polymorphism in the *Nlrp3* gene is closely associated with the colitis severity and progression in patients with IBD (111, 112). In addition, studies in macrophages and mice models of IBD have uncovered the link between the abnormal activation of the NLRP3 inflammasome and colitis (113, 114). It was recently reported that oral administration of titanium dioxide (TiO_2_) nanoparticles aggravated the severity of IBD through a mechanism involving the activation of the NLRP3 inflammasome in DSS-induced colitis mice or UC patents (108). Studies have reported that the induction of macrophage autophagy produced an inhibitory effect on the activation of the NLRP3 inflammasome in different kinds of inflammation-related diseases, including multiple sclerosis and several kinds of cardiovascular diseases (115, 116). Impairing autophagy by *Atg5* siRNA or 3-MA induced more robust initiation and activation of the NLRP3 inflammasome combined with increased caspase-1 activation and IL-1β production in peritoneal macrophages treated by LPS/DSS. Furthermore, using 3-MA to inhibit autophagy *in vivo* aggravated symptoms of DSS-induced colitis (117). Taken together, those results indicate that macrophage autophagy contributes to the alleviation of IBD through the inhibition of the NLRP3 inflammasome activation (illustrated in Figure 2). However, although the NLRP3 inflammasome has been shown to be highly related to the pathogenesis and progression of IBD and the crosstalk between autophagy and the NLRP3 inflammasome may probably provide a potential and promising therapeutic target for the treatment of IBD, yet the underlying mechanisms of IBD are not completely clear, and there is still a long way to go.

### Goblet Cell, IBD, and Autophagy

#### Goblet Cell and IBD

Goblet cell is another critical kind of intestinal epithelial cell which is derived from the stem cells located in the crypt bottom. The main function of goblet cell is storage and secretion of large amount of mucin granules containing mucin glycoproteins like mucin 2, antimicrobial factors, and mucus cross-linking proteins like Fc-gamma binding protein, which combine with intestinal epithelium to form the first line of defense against gut flora from the host (118, 119). Since goblet cells are the most highly secretory cells among all kinds of intestinal epithelial cells, the increased mucins misfolding or decreased mucin glycoproteins secretion can induce ER stress and unfolded protein response. Both mucins depletion and ER stress give rise to the activation of inflammatory and immune responses and subsequent intestinal inflammation in mice (120–122). Consistently, dysfunction in mucins secretion and defective in mucus layer allow large quantities of bacteria to reach the epithelium and triggering excess host immune responses which has been demonstrated to be associated with IBD (123).

#### The Roles of Goblet Cell Autophagy in IBD

Recently, it has been revealed that autophagy could control the secretion function of goblet cells (illustrated in Figure 1C). Mice or cells deficiency in autophagy by depletion of autophagy-related proteins like ATG5, ATG7, or LC3 led to altered goblet cell morphology and significantly decreased mucins secretion (124, 125). In addition, mice expressing the *ATG16l1 T300A* variant exhibited the similar characters of goblet cells (126). Patel et al. found out that nicotinamide adenine dinucleotide phosphate oxidase-driven ROS production was required for autophagy-mediated secretion of goblet cells as *Atg5*-deficient spheroids produced significantly diminished ROS compared with those wild type, and these cells were overloaded with mucins. Furthermore, goblet cells from mice treated with ROS scavenger *N*-acetylcysteine were accumulated with mucins compared to controls (124). The accumulated mucins in goblet cells suggested dysfunction in granules exocytosis. The upstream pathway inducing autophagy in goblet cells is mainly through activation of the NLRP6 inflammasome, a member of the Nod-like receptor family of PRRs. The NLRP6 inflammasome promotes goblet cell mucin granules exocytosis by promoting autophagy and goblet cells from *Nlrp6*-deficient mice have impaired autophagy and are less effective in secreting mucins. This secretion alteration leads to significant impaired role of epithelial barrier in colonic host–microbial interactions (127, 128). These findings suggest that defected autophagy in goblet cells contribute to the pathogenesis of IBD. Regulating autophagy in goblet cells may provide new strategies for the treatment of IBD. However, since the precise mechanisms and molecules involving autophagy in regulating mucins secretion are not fully elucidated, further studies are needed for the final clarification.

### The Roles of Autophagy in Colorectal Cancer (CRC)

Since IBD is likely to develop into CRC in the end and autophagy plays a pivotal role in the pathogenesis of IBD as discussed above, it is quite necessary to pay attention to the effects of autophagy on colorectal carcinogenesis. Autophagy is regarded as a double-edged sword during tumor progression, which suppresses tumor formation through inhibiting DNA injury, maintaining chromosomal stability, reducing local inflammation, and promoting immune system to eliminate potentially tumorigenic cells (129). However, on the other hand, autophagy can promote cancer cell survival and proliferation by escaping from intracellular and environmental stress as well as resisting to anticancer agents (129). Hence, the relationship between autophagy and CRC is complex. The first aspect lies in the expression profile of autophagy in CRC. Some evidence has shown that autophagy is highly induced in most CRC patients and CRC cells (130–135). However, there is also a small population of CRC patients with downregulated autophagy (136). Based on those reports, the expression profile of autophagy is inconsistent in CRC patients.

The second aspect is the complexity of the roles of autophagy in CRC. It was previously reported that overexpression of Beclin-1 *in vitro* inhibited CRC cells growth and enhanced the rapamycin-induced antitumor effects (136). Patients with high levels of Beclin-1 presented good prognosis and longer survival (131, 135), and ATG10 downregulation was associated with cell migration and invasion of CRC cells (137). In addition, several compounds were recently demonstrated to suppress colorectal carcinogenesis through the induction of autophagy, including Salvianolic acid B and IR-58 (138, 139). Those findings indicate that promoting autophagy may probably provide an effective strategy for the treatment of CRC. However, enhancing autophagy is not without its problems in the treatment of CRC. It was reported that promoting autophagy could enhance CRC cells tolerance to antitumor drugs (140, 141). For example, patients with high levels of Beclin-1 resist 5-FU-based adjuvant therapy and result in a poor prognosis and metastasis (142, 143). In addition, suppressing autophagy by deletion of *Atg5* enhanced the sensitivity of CRC cells and CRC mice to oxaliplatin and INF-γ (144, 145). In conclusion, since the functions of autophagy in CRC are complicated, to ultimately develop autophagy as therapeutic strategy in the treatment of CRC, numerous studies are needed in seek of effective approaches to induce autophagy while getting rid of its side effects.

## Pharmacological Intervention of Autophagy in the Treatment of IBD

In consideration of the importance of autophagy in the pathagenesis and progression of IBD, numerous studies have been conducted so far, focusing on the alleviation and treatment of IBD through the regulation of autophagy.

### Herbal Extracts

Andrographolide belongs to diterpenoids extracted from a kind of herb called andrographolide. Andrographolide has anti-inflammatory and antitumor activity (146, 147), and it can effectively inhibit the growth of breast and colon cancer cells (148). The combination of andrographolide and radiation therapy can enhance the levels of apoptosis and autophagy in nude mice xenografted by human ovarian cancer skov3 (149). It was reported that andrographolide sulfonate could inhibit the p38 mitogen-activated protein kinase (p38 MAPK) and NF-κB signaling pathways, which contributed to the reduction of pro-inflammatory cytokines production in trinitrobenzene sulfonic acid (TNBS)-induced colitis mice model and further intervened the progression of colitis (150). In addition, Guo et al. (151) demonstrated that andrographolide could enhance mitophagy in macrophages, lead to the reverse of mitochondrial membrane potential, and thus alleviate the symptoms of DSS-induced colitis through the inhibition of NLRP3 inflammasome activity. However, several autophagy inhibitors, including 3-MA, chloroquine, bafilomycin A1, or *Becline 1* siRNA abolished the inhibitory effects of andrographolide on the NLRP3 inflammasome and to a large extent reduced the therapeutic effects of andrographolide for colitis.

Another herbal extract worth mentioning is celastrol. It is a kind of triterpenoid extracted from the root of a traditional Chinese medicine called *Tripterygium wilfordii*. It was reported that celastrol could inhibit the development of IBD or colitis through several pathways, including: (1) regulating the oxidative stress and reducing lipid peroxide; (2) inhibiting the activation of the NLRP3 inflammasome; (3) increasing the levels of anti-inflammatory cytokines like IL-10; (4) enhancing the stability of intestinal epithelial barrier (152); (5) inhibiting the receptor interacting-protein 3/mixed lineage kinase domain-like protein-mediated programed necrosis pathway (153); and (6) inhibiting the production of NF-κB and its related inflammatory cytokines (154). Furthermore, it has been proven that celastrol can enhance the level of autophagy through the inhibition of the PI3K/Akt/mTOR signaling pathway in the colon tissue, and meanwhile reduce the production of several pro-inflammatory cytokines thus attenuating the inflammatory reaction in colon in IL-10-deficient mice (155). Based on those findings, it is reasonable to believe that those two herbal extracts may provide promising and effective therapies for IBD. However, since the clinical data for the treatment of IBD are poor, more detailed clinical studies are needed to explore their effectiveness and safety on IBD patients.

### Receptor Regulators

As been discussed above, the activation of the NLRP3 inflammasome leads to the activation of caspase-1, which induces the transformation of proIL-1β and proIL-18 into the mature IL-1β and IL-18. The maturation of IL-1β and IL-18 contributes to the pathogenesis and progression of inflammatory diseases. Studies indicated that autophagy play an important role in the formation and activation of NLRP3 inflammasome, and autophagy deficiency led to the abnormal increase of IL-1β (156–158). Mice deficient in autophagy showed more severe symptoms of colitis (59). Recently, increasing studies are conducted to explore whether blocking IL-1β contributes to alleviating the progression of IBD. For example, De Luca et al. (159) found out that the blockade of IL-1β receptor using anakinra could effectively alleviate the progression of IBD through the reduction of neutrophils accumulation and Th17 cell response. Van De Veerdonk and Dinarello (49) demonstrated that anakinra could restore the autophagy level of patients with chronic granuloma and reduced the IL-1-mediated inflammatory reaction, thus playing a protective role in the TNBS-induced colitis and alleviating the severity of patients with chronic granuloma, promoting the recovery of rectal abscess.

Cannabinoid receptor 2 (CB2R) is a kind of seven transmembrane-spanning G protein coupled receptor, mainly distributing on immune cells (160). Activating CB2R has been reported to contribute to the alleviation of EAE through the increase of autophagy, which in turn led to the inhibition of the NLRP3 inflammasome (115). Nowadays, the role of CB2R in IBD or colitis has been widely recognized. For example, it has been reported that activating CB2R can attenuate the severity of colitis in wild-type mice (161). In addition, activating CB2R was also demonstrated to alleviate colitis through inhibiting T cell activation and promote apoptosis in IL-10-deficient mice (162). In the recent years, several kinds of CB2R activators have been successfully applied in the treatment of colitis animal model (117, 163, 164). For example, activating CB2R by HU-308, a selective CB2R agonist, contributed to the enhancement of macrophage autophagy through the AMPK-mTOR-P70S6K signaling pathway, and further inhibited the NLRP3 inflammasome activation, thus reducing the level of IL-1β and alleviating the progression of DSS-induced colitis in mice (117) (illustrated in Figure 2). So far, although studies uncovered the promising therapeutic value of CB2R activators in the treatment of IBD, there is still no related drug taking advantage of the beneficial effects of activating CB2R for IBD. Hence, further studies are demanded for the development of related drugs and in the other hand, more kinds of receptor regulators should be fully studied to serve for the treatment of IBD.

### Nutrient Molecules

Vitamin D deficiency is regarded as an important pathological basis of IBD (165). Studies showed that the level of vitamin D is relatively low in patients with IBD (166, 167). Vitamin D leads to a series of physiological effects mainly through functioning on VDR, of which the gene polymorphism is closely connected with the susceptibility of IBD (168). It was reported that deficiency in vitamin D can lead to the reduction of ATG16L1 and the level of autophagy and further influenced the function of Paneth cells. After restoring the level of VDR by applying vitamin D3 in intestinal epithelial cells, the levels of autophagy is increased (78). Furthermore, a systematic review and meta-analysis conducted by Ma et al. (169) demonstrated that vitamin D intake as well as blood 25(OH)D levels were inversely associated with the risk of CRC. In consideration of the closely relationship between IBD and CRC, the antitumor feature of vitamin D provides further evidence to show the therapeutic value of vitamin D in the treatment of IBD.

Glutamine is a kind of free amino acid widely distributed in organisms. It is the respiratory material and metabolic precursors of many kinds of cells, including intestinal epithelial cells and immune cells (170–172). Under the physiological stress, glutamine reduces the permeability of intestinal epithelial cells, inhibits intestinal mucosa injury, and promotes the intestinal restoration (173–175). It was reported that in the intestinal epithelial cells glutamine contributed to the enhancement of autophagy level in the basal and stressing condition through the regulation of the mTOR and p38 MAPK signaling pathway, thus inhibiting the stress-induced cellular apoptosis (176).

### Other Small-Molecular Compounds

Two kinds of rapamycin analogs, namely, sirolimus and everolimus, which have already been applied in clinic, contribute to the enhancement of autophagy level. It has been reported that continuous application of sirolimus for 6 months significantly led to the attenuation of symptoms in patients with severe recurrent CD (177). Sirolimus can not only benefit adult IBD patients, but also lead to the alleviation of IBD in the children patients. It was reported that after the application of sirolimus, 45% UC patients and 100% CD patients reached the clinical stable stage. Meanwhile, sirolimus contributes to the promotion of intestinal mucus restoration (178). In addition, after the occurrence of the spontaneous colitis in IL-10-deficient mice, researchers found out that treating with everolimus for 4 weeks significantly reduced the disease activity index as well as the infiltration of lymphocytes in spleen, mesenteric lymph nodes, and inherent layer (179). It was lately reported by Dumortier et al. (180) that after treated with everolimus in the original treatment therapy for 1½ years, the symptoms in a patient with refractory UC were effectively controlled. However, in a multicenter randomized double bind trial, everolimus was not beneficial for the maintenance of the stable stage treated with glucocorticoid in patients with activity stage in the present of placebo or azathioprine (181). This study indicates that the therapeutic strategy for IBD through enhancing autophagy process demands further assessment and the effectiveness and security needs further tested.

The drugs or molecules mentioned above that alleviate IBD through enhancing autophagy were listed in the Table 1, and some other drugs were also briefly mentioned.

**Table 1:** **The mechanisms of autophagy inducers in treatment of IBD**.

Category	Name	Mechanism	Reference
Herbal extract	Andrographolide	Inhibits NLRP3 inflammasome	(151)
	Celastrol	Reduces pro-inflammatory cytokines production	(155)
Receptor regulator	Interleukin-1β receptor blocker	Reduces neutrophils accumulation and Th17 cell response	(49, 159)
	CB2 receptor agonist	Inhibits NLRP3 inflammasome	(117)
	Nicotine	Increases cyclooxygenase-2 and prostaglandin E2	(182)
Nutrient molecular	Vitamine D3	Restores vitamin D receptor level	(78, 183, 184)
	Glutamine	Inhibits cellular apoptosis	(176)
	Docosahexaenoic acid	Inhibits the mTOR signaling pathway	(185)
Other small-molecular compound	Sirolimus	Promotes intestinal mucus restoration	(177, 178)
	Everolimus	Reduce lymphocytes infiltration	(179, 180)

## Conclusion

Intestinal autophagy, especially autophagy in Paneth cells, macrophages, and goblet cells in the intestinal wall, produces an alleviative effect on the pathogenesis and progression of IBD. In the recent few years, we are pleased to have several kinds of drugs developed for the treatment of IBD taking advantage of the induction of autophagy, including herbal extracts, receptor regulators, nutrient molecules, and other small-molecular compounds, etc. However, despite the numerous therapeutic strategies available, there is still no effective way for the treatment of IBD. Furthermore, the specific mechanism of IBD still remains uncovered. Besides, there are several other hot-discussed issues worth mentioning in this field: first, since little literatures have reported the effects of autophagy inducers in the clinical treatment of IBD, proper doses demand further testing and side effects ought to be found out for the safe application. Second, based on the discussion above, although inducing autophagy contributes to the alleviation of IBD, yet it has already been reported that on certain conditions, the induction of autophagy may also lead to inverse effects on the attenuation of IBD (186–188). Third, as discussed above, although inducing autophagy contributes to the suppression of colorectal carcinogenesis, there are still side effects that autophagy inducers may lead to the enhancement of CRC cells tolerance to antitumor drugs. Therefore, more studies are needed to explore the mechanisms of IBD, and effective therapeutic strategies are demanded to be developed while getting rid of side effects of inducing autophagy.

## Author Contributions

PK, B-ZS, and Z-QX retrieved and analyzed concerned literatures. PK and B-ZS wrote the manuscript. CL and X-WC revised the manuscript. All the authors agreed to be accountable for the content of the work.

## Conflict of Interest Statement

The authors declare that the research was conducted in the absence of any commercial or financial relationships that could be construed as a potential conflict of interest.
